# Phenotypic and genetic characterization of β-lactam resistance in *Klebsiella* from retail chicken meat in Mansoura, Egypt

**Published:** 2017-04

**Authors:** Hazem Ramadan, Amal Awad

**Affiliations:** 1 Hygiene and Zoonoses Department, Faculty of Veterinary Medicine, Mansoura University, Mansoura 35516, Egypt; 2 Bacteriology, Mycology and Immunology Department, Faculty of Veterinary Medicine, Mansoura University, Mansoura 35516, Egypt

**Keywords:** *Klebsiella*, Retail chicken meat, Antimicrobial resistance, ESBL, Food-borne pathogens

## Abstract

**Background and Objectives:**

This study was undertaken to characterize antimicrobial resistance phenotypes and genes encoding extended spectrum β-lactamases (ESBLs) in *Klebsiella* isolated from retail chicken meat in Mansoura, Egypt.

**Materials and Methods:**

Three hundred sixty chicken meat samples from 120 eviscerated chicken carcasses (3 cuts each) collected randomly from local retail chicken shops in Mansoura, Egypt during the period from April to June 2015, were assayed for the presence of *Klebsiella* by conventional bacteriological methods. Antimicrobial sensitivity for 12 antimicrobials using disk diffusion, ESBL phenotypic confirmation and PCR characterization of ESBL-encoding genes (*bla*_ TEM_ , *bla*_ CTX-M_ , *bla*_ OXA_ , *bla*_ SHV_ and *bla*_ CMY _) were performed.

**Results:**

*Klebsiella* was identified from 22.2% (80/360) of the samples. Of the 12 antimicrobials tested, multidrug resistance (MDR; resistance to ≥3 of the antimicrobial classes) was observed in 96.25% (77/80) of the *Klebsiella* isolates. All the isolates were resistant to cefotaxime, ceftriaxone and aztreonam. ESBL-producers were phenotypically confirmed in 48.75% (39/80) of the isolates. The highest values (0.75 and 0.67) of multiple antibiotic resistance (MAR) significantly occurred in ESBL-producing isolates. PCR findings showed a significantly higher occurrence of β-lactamase encoding genes in ESBL (94.9%, 37/39) than non-ESBL producing isolates (4.9%, 2/41). The distribution of *bla*_ TEM_ , *bla*_ CTX-M_ and *bla*_ OXA_ among ESBL-producing isolates was 84.6%, 30.8% and 25.6%, respectively.

**Conclusion:**

Efficient monitoring and tracking of MDR, especially β-lactam resistance, in food sources is essential to predict the potential hazards for human infections.

## INTRODUCTION

The unrestricted access and the overuse of anti-microbial drugs in animals to control bacterial infections has led to the emergence of multidrug resistance among pathogenic bacteria. Additionally, administration of these antimicrobials as growth promoters in chicken farms when fed at sub-therapeutic doses has complicated the problem of antimicrobial resistance (1). Multidrug resistance, particularly extended spectrum β-lactamase (ESBL) resistance, has increased over the last two decades among Enterobacteriaceae especially *Escherichia coli* and *Klebsiella* that constitutes challenges to public health (2, 3).

*Klebsiella* species are classified as one of the opportunistic bacteria that normally inhabit the gastrointestinal tract of healthy humans and animals, and are also commonly found in the environment (4). Reports by Kim et al. (5) and Price et al. (6) revealed that occupational contact with contaminated environmental sources affords a possible way for *Klebsiella* to infect humans. Moreover, chicken meat has been considered another source for human infections with multidrug resistant *Klebsiella* (7–9). Chicken meat has been contaminated most likely from the chicken gut if the process of slaughtering and evisceration occurred improperly (10).

ESBL-producing bacteria secret enzymes which are encoded by β-lactamases, responsible for the hydrolysis of β-lactam ring in penicillins, cephalosporins and monobactams (11). The most common β-lactamase genes that encode ESBL activity are *bla*_ TEM_ , *bla*_ SHV_ , *bla*_ CTX-M_ , and *bla*_ OXA_ which have been detected globally (12). ESBL genes are located on plasmids with a possibility for horizontal transmission between strains of the same species or different bacterial genera (13).

To minimize antimicrobial resistance in poultries, many regulations have been established in developed countries. These measures are mainly concerned with the restriction of antimicrobial usage and establishing a rigorous surveillance and monitoring system of antimicrobial resistance inside farms (14). However, the problem is drastically increased in developing countries such as Egypt (15) with a consequent public health concern.

This study aimed to determine antimicrobial resistance phenotypes and genes encoding ESBLs in *Klebsiella* isolated from retail chicken meat in Mansoura, Egypt. This data can provide useful information that might aid the management of antimicrobial therapies in poultries.

## MATERIALS AND METHODS

### Sampling and bacterial isolation.

A total of 360 chicken meat samples from 120 eviscerated chicken carcasses (3 cuts each per chicken carcass including the back, breast and thigh) were collected randomly from local grocery stores and chicken retail shops in Mansoura, Egypt during the period from April to June 2015. Each sample was individually packaged in sterile polyethelene bags and transferred in ice tanks to the Bacteriology, Mycology and Immunology Laboratory, Faculty of Veterinary Medicine, Mansoura University for bacteriological examination under aseptic conditions.

A cubic piece of 25 g from each chicken meat cut was homogenized into 225 ml of nutrient broth (Becton Dickinson, Sparks, MD, USA) and incubated overnight at 37°C. A loopful from the enriched broth mixture was inoculated onto the surface of MacConkey agar plates (Oxoid, UK) and incubated at 37°C for 1–2 days. Morphological characterization of the suspected *Klebsiella* colonies on MacConkey plates was performed as previously described (16). Further identification was performed by subjecting the purified presumptive *Klebsiella* colonies to different biochemical tests including oxidase, catalase, indole production, Voges-Proskauer, methyl red, citrate utilization, lysine decarboxylase, lactose fermentation, urea hydrolysis, and H _2_ S production (17).

### Antimicrobial susceptibility testing.

All recovered isolates were phenotypically tested using the Kirby-Bauer disk diffusion method against 12 antimicrobials that belonged to 7 antimicrobial classes. Resistance was determined according to the Clinical and Laboratory Standards Institute (CLSI) guidelines (18). The antimicrobials (Oxoid, UK) tested were as follows: amoxicillin (Ax; 30 μg), piperacillin (PRL; 100 μg), norfloxacin (NOR; 10 μg), ciprofloxacin (CIP; 5 μg), amikacin (AK; 30 μg), neomycin (N; 30 μg), chloramphenicol (C; 30 μg), cefepime (FEP; 30 μg), ceftriaxone (CRO; 30 μg) cefotaxime (CTX; 30 μg), aztreonam (ATM; 30 μg) and amoxicillin/clavulanic acid (AMC; 30 μg). The multiple antibiotic resistance (MAR) index was determined by dividing the total number of resistances to antimicrobials for each isolate to the total number of tested antimicrobials (19).

### ESBL confirmatory test.

The double disk diffusion assay was used for the confirmation of the ESBL phenotype according to the standard criteria of CLSI (18). All isolates identified in this study were tested against cefotaxime (CTX; 30 μg) and ceftazidime (CTZ; 30 μg) disks with and without 10 μg clavulanic acid (CA) (Oxoid, UK). The isolate was considered an ESBL-producer when it exhibited an increase in the zone of inhibition by a 5 mm or more diameter with the combined disc containing cephalosporin plus CA compared to the disk containing cephalosporin alone.

### DNA extraction and molecular identification of ESBL genes.

From each MacConkey plate, three to five representative colonies of similar morphological pattern were selected for genomic DNA extraction by boiling as previously described by Ramadan et al. (20). The purified DNA extracts were aseptically transported to the Central Diagnostic and Research Laboratory, Faculty of Veterinary Medicine, Kafrelsheikh University, for the molecular identification of ESBL genes.

Primer pairs for *bla*_ TEM_ , *bla*_ CTX-M_ , *bla*_ OXA_ , *bla*_ SHV_ and *bla*_ CMY_ are listed in Table 1 (21, 22). The cyclic conditions of the two uniplex PCRs targeting *bla*_ TEM_ and *bla*_ SHV_ were performed as previously described (23). Amplification of *bla*_ OXA_ , *bla*_ CTX-M_ and *bla*_ CMY_ was done in three uniplex PCRs according to Siu et al. (24), Zhao et al. (21) and Ahmed et al. (25), respectively with minor modifications. The amplified PCR products for each gene were electrophoresed in 1.5% agarose for 50 min at 90 V, stained with ethidium bromide and visualized under ultraviolet light. DNA extracts from previously identified ESBL-producing *E. coli* were kindly provided by Central Diagnostic and Research Laboratory, Faculty of Veterinary Medicine, Kafrelsheikh University and used as positive controls.

**Table 1.** List of the primers used for the characterization of ESBL genes in *Klebsiella* isolates from retail chicken meat.

**Primer sequence**	**Target gene**	**Target amplicon**	**Reference**
5′-GACAGCCTCTTTCTCCACA-3′	*bla*_CMY_.F	1007 bp	(21)
5′-TGGAACGAAGGCTACGTA-3′	R		
5′ATAAAATTCTTGAAGACGAAA-3′	*bla*_TEM_.F	1080 bp	(22)
5′-GACAGTTACCAATGCTTAATC-3′	R		
5′CGCTTTGCGATGTGCAG-3′	*bla*_CTX-M_.F	550 bp	(22)
5′-ACCGCGATATCGTTGGT-3′	R		
5′-TCAACTTTCAAGATCGCA-3′	*bla*_OXA_.F	591 bp	(22)
5′-GTGTGTTTAGAATGGTGA-3′	R		
5′-TTATCTCCCTGTTAGCCACC-3′	*bla*_SHV_.F	795 bp	(22)
5′-GATTTGCTGATTTCGCTCGG-3′	R		

### Statistical analysis.

Chi-square (χ^2^) test was performed to determine the statistical association of multidrug resistance within ESBL and non-ESBL-producing *Klebsiella* at a probability value p < 0.05 using SPSS version 16.0 (SPSS Inc., Chicago, USA) software.

## RESULTS

In this study, a total of 80 presumptive *Klebsiella* isolates were identified from the examined chicken meat samples with an overall prevalence of 22.2%. All recovered isolates were phenotypically tested against 12 antimicrobials and variable resistance patterns were detected (Table 2). All *Klebsiella* isolates from this study exhibited resistance against cefotaxime, ceftriaxone and aztreonam.

**Table 2.** Antimicrobial resistance/sensitivity percentages in *Klebsiella* isolates from retail chicken meat.

**Antimicrobial agents tested**	**Disc code**	**Antimicrobial class**	**Resistance**		**Sensitivity**	
			**No.**	**%**	**No.**	**%**
Amoxicillin	AX	Penicillins	77	96.25%	3	3.75%
Piperacillin	PRI	Penicillins	74	92.5 %	6	7.5%
Norfloxacin	NOR	Quinolones	30	37.5%	50	62.5%
Ciprofloxacin	CIP	Quinolones	32	40%	48	60%
Amikacin	AK	Aminoglycosides	0	0	80	100%
Neomycin	N	Aminoglycosides	0	0	80	100%
Chloramphenicol	C	Phenicols	15	18.75%	65	81.25%
Cefepime	FEP	Cephalosporins	52	65%	28	35%
Ceftriaxone	CRO	Cephalosporins	80	100%	0	0
Cefotaxime	CTX	Cephalosporins	80	100%	0	0
Aztreonam	ATM	Monobactams	80	100%	0	0
Amoxicillin /clavulanic acid	AMC	β-Lactam/ β-lactamase inhibitors	42	52.5%	38	47.5%

Resistance among the *Klebsiella* isolates to amoxicillin, piperacillin, cefepime and amoxicillin/clavulanic acid was 96.25, 92.5%, 65% and 52.5%, respectively. Of the two quinolones tested, 37.5% (30/80) and 40% (32/80) of the examined isolates showed resistance to norfloxacin and ciprofloxacin, respectively. All isolates exhibited 100% susceptibility against the two aminoglycosides (amikacin and neomycin) tested in this study.

Multidrug resistance to ≥ 3 of the antimicrobial classes tested was detected in 77 (96.25%) of the *Klebsiella* isolates. A total of 14 and 22 isolates had the antimicrobial resistance patterns (AX, PRI, CTX, CRO, ATM, AMC) and (AX, PRI, FEP, CTX, CRO, ATM, AMC), respectively. Furthermore, 21 (26.3%) *Klebsiella* isolates exhibited multidrug resistance to at least 5 antibiotic classes. These 21 isolates were separated into 2 antimicrobial resistance patterns, (AX, PRI, NOR, CIP, C, FEP, CTX, CRO, ATM) (15/21, 71.4%) and (AX, PRI, NOR, FEP, CTX, CRO, ATM, AMC) (6/21, 28.6%). Of the 80 *Klebsiella* isolates, 39 (48.75%) were identified phenotypically as ESBL-producers. The prevalence of the highest MAR indices (0.75 and 0.67) was significantly higher in ESBL than non-ESBL-producing *Klebsiella* isolates (Table 3).

**Table 3.** The distribution of antimicrobial resistance profiles among ESBL and non ESBL-producing *Klebsiella* isolates from retail chicken meat.

**Antimicrobial resistance profile (phenotype)**	**No. of isolates**	**MAR index**	**ESBL-producer No. (%)**	**Non ESBL-producer No. (%)**
AX, PRI, NOR, CIP, C, FEP, CTX, CRO, ATM	15	0.75	11 (73.3%)^a^	4 (26.7%)^b^
AX, PRI, NOR, FEP, CTX, CRO, ATM, AMC	6	0.67	6 (100%)^a^	0^b^
AX, PRI, NOR, CIP, FEP, CTX, CRO, ATM	9	0.67	6 (66.7%)	3 (33.3%)
AX, PRI, FEP, CTX, CRO, ATM, AMC	22	0.58	7 (31.8%)	15 (68.2%)
AX, PRI, CTX, CRO, ATM, AMC	14	0.5	2 (14.3%)^a^	12 (85.7%)^b^
AX, PRI, CIP, CTX, CRO, ATM	8	0.5	1 (12.5%)^a^	7 (87.5%)^b^
AX, CTX, CRO, ATM	3	0.33	3 (100%)	0
CTX, CRO, ATM	3	0.25	3 (100%)	0

Different superscripts within each row indicate significant differences (p < 0.05).

**Fig. 1.**
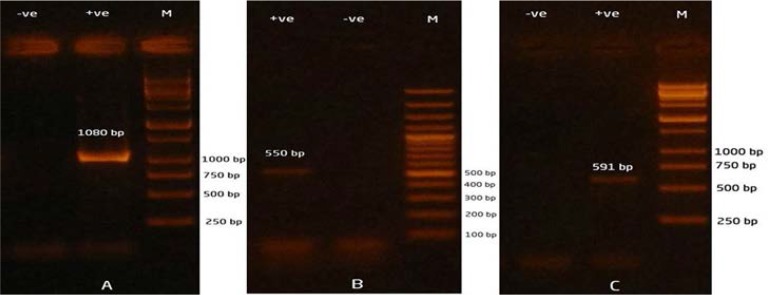
Gel electrophoresis of the uniplex PCR amplifications of A) *bla*_TEM_ gene, B) *bla*_CTX-M_ gene and C) *bla*_OXA_ gene in *Klebsiella* isolates from retail chicken meat. Lane M: 100 bp DNA ladder for *bla*_CTX-M_ and 1000 bp DNA ladder for both *bla*_TEM_ and *bla_OXA_* genes. Lane −ve: negative control. Lane +ve: positive control.

All *Klebsiella* isolates were screened using five uniplex PCRs for the identification of ESBL genes (*bla*_ TEM_ , *bla*_ CTX-M_ , *bla*_ OXA_ , *bla*_ SHV_ and *bla*_ CMY _). In ESBL-producing isolates, 94.9% (37/39) carried at least one β-lactamase encoding genes (Table 4). For the ESBL-producing isolates, *bla*_ TEM_ , *bla*_ CTX-M_ and *bla*_ OXA_ was found in 84.6% (33/39), 30.8% (12/39) and 25.6% (10/39) of isolates, respectively (Fig. 1). No isolates were positive for *bla*_ SHV_ and *bla*_ CMY_ ; only 4.9% (2/41) of the non-ESBL-producing isolates contained *bla*_ OXA_ .

**Table 4.** Antimicrobial resistance profiles and resistance genes pattern in ESBL-producing *Klebsiella* isolates.

**No. of strains**	**Antimicrobial resistance profile (phenotype)**	**ESBL encoding genes pattern**
7	AX, PRI, FEP, CTX, CRO, ATM, AMC	*bla*_TEM_
3	AX, PRI, NOR, FEP, CTX, CRO, ATM, AMC	*bla*_TEM_
5	AX, PRI, NOR, CIP, C, FEP, CTX, CRO, ATM	*bla*_TEM_
6	AX, PRI, NOR, CIP, C, FEP, CTX, CRO, ATM	*bla*_TEM_+*bla*_CTX-M_
2	AX, PRI, NOR, CIP, FEP, CTX, CRO, ATM	*bla*_TEM_+*bla*_CTX-M_
1	AX, PRI, CIP, CTX, CRO, ATM	*bla*_TEM_+*bla*_CTX-M_
3	AX, PRI, NOR, FEP, CTX, CRO, ATM, AMC	*bla*_TEM_+*bla*_CTX-M_
2	AX, PRI, CTX, CRO, ATM, AMC	*bla*_TEM_+*bla*_OXA_
2	AX, CTX, CRO, ATM	*bla*_TEM_+*bla*_OXA_
2	AX, PRI, NOR, CIP, FEP, CTX, CRO, ATM	*bla*_TEM_+*bla*_OXA_
1	AX, CTX, CRO, ATM	*bla*_OXA_
1	CTX, CRO, ATM	*bla*_OXA_
2	AX, PRI, NOR, CIP, FEP, CTX, CRO, ATM	*bla*_OXA_
2	CTX, CRO, ATM	ND^*^

ND^*^, Not detected.

## DISCUSSION

The current study was performed to determine antimicrobial resistance, especially β-lactam resistance, among *Klebsiella* isolates from retail chicken meat. Many recent studies have shown increasing prevalence of antimicrobial resistance among *Klebsiella* isolates from chicken meat with a potential public health threat (9, 26–28). The highest level of antimicrobial resistance was observed against β-lactams, particularly cephalosporins, monobactams and penicillins. This was in agreement with data previously reported by Wu et al. (29). Variable percentages of resistance to third generation cephalosporins have been determined for *Klebsiella* by Ullah et al. (30) (54.4%) and Gundogan et al. (31) (11%). A recent study in China showed that 96% of the *Klebsiella* isolates from raw chicken samples exhibited resistance to ampicillin and only 3.8% of the isolates were resistant to cefotaxime (9).

The increased prevalence of antimicrobial resistance in this study to amoxicillin (96.25%) and piperacillin (92.5%) was not unexpected due to the natural existence of the chromosomal class A β-lactamase which is responsible for penicillin resistance in most *Klebsiell*a isolates (32).

The lack of susceptibility in approximately two-thirds of the *Klebsiella* isolates to cefepime, a fourth generation cephalosporin, is alarming. Lower percentages of antimicrobial resistance to cefepime were detected by Gundogan and Avci (27) (0%) and Guo et al. (9) (3.8%). The increased resistance in this study could be linked to the inappropriate introduction of cefepime into poultry farms for controlling infections caused by Gram-negative bacteria especially those exhibiting multidrug resistance to third generation cephalosporins.

Approximately, one-third of the isolated *Klebsiella* from chicken meat exhibited resistance against both norfloxacin and ciprofloxacin. In Egypt, variable antimicrobial resistance frequencies to quinolones have been recorded among Enterobacteriaceae. Ahmed and Shimamoto (33) and Halawa et al. (34) reported that 47% and 8.7% of the isolated *Salmonella* from diseased broilers conferred resistance to ciprofloxacin. Also, 15.1% of the isolated avian pathogenic *E. coli* from septicemic broilers displayed resistance to ciprofloxacin (35). This provides an indication about the continuous administration of quinolones in poultry farms in Egypt.

No resistance has been determined for *Klebsiella* to aminoglycoside antibiotics including amikacin and neomycin. This was in agreement with that recorded previously by Guo et al. (9) who found that all the *Klebsiella* isolates from different food samples were susceptible to amikacin. Different resistance rates to amikacin were detected in previous reports. Ullah et al. (30) and Gundogan et al. (31) reported that 37% and 7% of the *Klebsiella* isolates were susceptible to amikacin in the North West of Pakistan and Turkey, respectively.

The results of the present study showed that 96.25% of the strains possessed multidrug resistance. Nearly similar results were previously determined in *Klebiella* isolates from different animal sources (29, 36). The above findings also showed that 48.75% of the isolated *Klebsiella* were ESBL-producers. The significantly higher occurrence of multidrug resistance in ESBL than non-ESBL-producing *Klebsiella* isolates was expected (31). ESBL-producing *Klebsiella* have been reported with different prevalences as determined by Gundogan et al. (31) (29%, 13/45), Overdevest et al. (37) (7.7%) and Wu et al. (36) (96.7%). The differences in the above percentages could be linked to many factors such as geographical and climatic differences, regime of infection control, farm biosecurity measures, drug administration and sampling technique.

All isolates were subjected to PCR for the identification of β-lactamase encoding genes (*bla*_ TEM_ , *bla*_ SHV_ , *bla*_ OXA_ , *bla*_ CTX-M_ and *bla*_ CMY _). Findings showed that 94.9% (37/39) and 4.9% (2/41) of ESBL and non-ESBL-producing isolates carried at least one β-lactamase encoding gene, respectively. Expectedly, there was a significant higher occurrence (χ ^2^ =64.791, *P*<0.05) of β-lactamase encoding genes in ESBL than non-ESBL-producing isolates. However, the presence of these genes in two non-ESBL-producing isolates could be attributed to the inability of these isolates to express their β-lactamase genes (36).

Our study demonstrated that the most prevalent β-lactamase gene detected was *bla*_ TEM_ in 84.6% (33/39) of the ESBL-producing isolates followed by *bla*_ CTX-M_ (30.8%, 12/39) and *bla*_ OXA_ (25.6%, 10/39). Many recent reports showed that retail chicken meat has been indicated as a potential source of bacteria containing β-lactamase encoding genes (9, 36, 38–40). Since most ESBL genes are plasmid-encoded, there is a possibility of transmission within the same bacterial species as well as among different bacterial genera (41, 42).Therefore, human acquisition of ESBL genes could be originating from the contaminated retail chicken meat with ESBL-producing *Klebsiella.*

In conclusion, our findings signify the contributing role of chicken meat as potential sources for human exposure and infections with ESBL-producing *Klebsiella* through the food chain. This study also recommends the implementation of monitoring systems and legalizations that prohibit the improper use of antibiotics which in turn will minimize the increasing incidence of multidrug resistance in Egypt. Future research will be aimed at determining phylogenetic relatedness between chicken and human *Klebsiella* isolates circulating in the study area.
